# Prevalence and risk factors for patient-reported joint pain among patients with HIV/Hepatitis C coinfection, Hepatitis C monoinfection, and HIV monoinfection

**DOI:** 10.1186/s12891-015-0552-z

**Published:** 2015-04-19

**Authors:** Alexis Ogdie, Wyki Gina Pang, Kimberly A Forde, Bhangle D Samir, Lakeisha Mulugeta, Kyong-Mi Chang, David E Kaplan, Valerianna K Amorosa, Jay R Kostman, Rajender K Reddy, Ralph H Schumacher, Vincent Lo Re

**Affiliations:** 1 Division of Rheumatology, Center for Clinical Epidemiology and Biostatistics, Perelman School of Medicine at the University of Pennsylvania, Penn Tower Room 1407, 1 Convention Ave, Philadelphia, PA 19104 USA; 2 Maine Medical Center, Tufts University School of Medicine, Portland, ME USA; 3 Department of Medicine, Division of Gastroenterology, Center for Clinical Epidemiology and Biostatistics, Perelman School of Medicine at the University of Pennsylvania, Philadelphia, PA USA; 4 Seacoast Arthritis and Osteoporosis Center, 10 Members Way, Suite 403, Dover, NH 03820 USA; 5 Perelman School of Medicine, the University of Pennsylvania, Philadelphia, PA USA; 6 Division of Gastroenterology, Philadelphia VA Medical Center, Perelman School of Medicine at the University of Pennsylvania, Philadelphia, PA USA; 7 Division of Infectious Diseases, Philadelphia VA Medical Center, Perelman School of Medicine at the University of Pennsylvania, Philadelphia, PA USA; 8 Division of Gastroenterology, Perelman School of Medicine at the University of Pennsylvania, Philadelphia, USA; 9 Division of Rheumatology, Philadelphia VA Medical Center, Perelman School of Medicine at the University of Pennsylvania, Philadelphia, PA USA; 10 Department of Medicine, Division of Infectious Diseases, Center for Clinical Epidemiology and Biostatistics, Perelman School of Medicine at the University of Pennsylvania, Philadelphia, PA USA

**Keywords:** Hepatitis c, HIV, Arthralgia, Epidemiology

## Abstract

**Background:**

To determine the prevalence of patient-reported joint pain among patients with human immunodeficiency virus (HIV)/chronic hepatitis C virus (HCV) coinfection, chronic HCV monoinfection, and HIV monoinfection followed in hepatology and infectious disease outpatient practices.

**Methods:**

Standardized interviews were performed among 79 HIV/HCV-coinfected, 93 HCV-monoinfected, and 30 HIV-monoinfected patients in a cross-sectional study within hepatology and infectious disease clinics at three centers. The Multi-Dimensional Health Assessment Questionnaire was used to ascertain joint pain and associated symptoms. Information on potential risk factors for joint pain was obtained during the interview and by chart review. Logistic regression was used to determine adjusted odds ratios (aORs) with 95% confidence intervals (CIs) of joint pain associated with risk factors of interest among chronic HCV-infected and HIV-infected patients.

**Results:**

Joint pain was more commonly reported in HCV-monoinfected than HIV/HCV-coinfected (71% versus 56%; p = 0.038) and HIV-monoinfected (71% versus 50%; p = 0.035) patients. A previous diagnosis of arthritis and current smoking were risk factors for joint pain among HCV-infected patients (arthritis: aOR, 4.25; 95% CI, 1.84-9.81; smoking: aOR, 5.02; 95% CI, 2.15-11.74) and HIV-infected (arthritis: aOR, 5.36; 95% CI, 2.01-14.25; smoking: aOR, 6.07; 95% CI, 2.30-16.00) patients.

**Conclusion:**

Patient-reported joint pain was prevalent among all three groups, but more common among chronic HCV-monoinfected than either HIV/HCV-coinfected or HIV-monoinfected patients. A prior diagnosis of arthritis and current smoking were risk factors for patient-reported joint pain among both HCV-infected and HIV-infected patients.

**Electronic supplementary material:**

The online version of this article (doi:10.1186/s12891-015-0552-z) contains supplementary material, which is available to authorized users.

## Background

Hepatitis C virus (HCV) infection is the most common blood-borne infection in the United States and is a leading cause of advanced liver disease. Over 4 million people in the U.S. and more than 170 million people worldwide have been infected with HCV infection [1,2]. Many extrahepatic manifestations have been associated with chronic HCV infection, including dermatologic, neurologic, renal, and rheumatic disorders [3-10]. While chronic HCV-induced inflammation is generally thought to be a key contributor to these manifestations, the mechanisms by which they occur remain unclear [11].

Rheumatologists are frequently faced with managing chronic HCV-infected patients with joint pain. However, few published studies have evaluated: 1) the prevalence of patient-reported joint pain among patients with chronic HCV in the U.S., 2) how joint symptoms among patients with chronic HCV affect quality of life and functional ability, or 3) whether coinfection with chronic HCV and human immunodeficiency virus (HIV) affects the prevalence of patient-reported joint pain. HIV infection has also been associated with rheumatic conditions, including reactive arthritis, psoriatic arthritis, and arthralgias and myalgias without identifiable inflammatory disease [12]. While HIV coinfection accelerates chronic HCV-related liver fibrosis progression to cirrhosis and hepatic decompensation, [13-15] the role of HIV/HCV coinfection on the development of joint pain remains unknown.

The primary objective of this study was to determine the prevalence of patient-reported joint pain (arthralgias) among patients with HIV/chronic HCV coinfection, chronic HCV monoinfection, and HIV monoinfection followed in hepatology and infectious disease clinics. We hypothesized that there would be a higher prevalence of arthralgias in HIV/HCV-coinfected patients compared to those who have chronic HCV or HIV alone. Our second objective was to examine potential risk factors for patient-reported joint pain among chronic HCV-infected (i.e., HIV/HCV-coinfected and HCV-monoinfected) and HIV-infected (i.e., HIV/HCV-coinfected and HIV-monoinfected) patients.

## Methods

### Study design and setting

We performed a cross-sectional study among patients with HIV/chronic HCV coinfection, chronic HCV monoinfection, and HIV monoinfection. Consecutive patients with chronic HCV and/or HIV infections were recruited in hepatology and infectious disease clinics at three tertiary care medical centers within Philadelphia (Penn Presbyterian Medical Center [PPMC], Philadelphia Veterans Affairs Medical Center [PVAMC], and the Hospital of the University of Pennsylvania [HUP]). The PPMC infectious disease outpatient practice specializes in viral hepatitis care; hepatologists and infectious disease physicians see patients concurrently in the HUP viral hepatitis clinic; and the PVAMC outpatient infectious disease and hepatology clinics are located within the same practice space. Patients were enrolled between November 2008 and March 2012.

### Study patients

Participants were eligible for inclusion if they were between 18 and 80 years of age and had documentation of HIV (HIV antibody- or RNA-positive) and/or chronic HCV infection (HCV RNA-positive) in their medical record. Exclusion criteria included the inability to either speak or understand English or to provide informed consent.

### Measurements

Participants were asked to complete an interviewer-administered questionnaire that ascertained the presence or absence of patient-reported joint pain over the one week prior to the study visit, the duration of joint pain, the joints affected, and previous diagnoses of joint disorders or arthritis. Among patients reporting joint pain, an interviewer administered the Multi-Dimensional Health Assessment Questionnaire (MD-HAQ, version R780-NP2) [16] given the highly varied reading skills and medical literacy in the patient population surveyed. Interviews were standardized among the four questionnaire administrators (WGP, LM, SB, and AO). The MD-HAQ includes a physical function score (range, 0 to 10), emotional function score (0 to 9.9), a pain score (range, 0 to 10), global health assessment (range, 0 to 10), fatigue scale (range, 0 to 10), ratings of painful joints, responses to a review of systems, and basic demographics (e.g. age, sex, height, weight, education, employment status and occupation). The MD-HAQ scores for physical function, pain, and global health status can be combined into a composite index known as the Routine Assessment of Patient Index Data (RAPID3; range, 0 to 30). In rheumatoid arthritis, a RAPID3 score of > 12 (on a scale from 0–30) indicates high disease activity, a score of 6.01-12 suggests moderate disease activity, a score of 3.01-6 suggests lower disease activity, and scores ≤ 3 indicate disease remission [17]. The MD-HAQ also includes three questions on “emotional function” (depression, anxiety, sleep disturbance), each ranging from 0 to 3.3 in 1.1 increments. These three scores were summed to result in an “emotional function” score ranging from 0–9.9. The MD-HAQ includes a review of systems, including symptoms such as dry mouth, dry eyes, numbness and tingling in the extremities, myalgias, and morning stiffness (a total of 60 symptoms are included in the review of systems) and a visual analog scale for fatigue. The MD-HAQ and RAPID3 have been validated in patients with rheumatoid arthritis and have previously been used in rheumatologic epidemiological studies [18-20]. Additionally, the MDHAQ has been used in clinical practice to evaluate many different diseases, including osteoarthritis and fibromyalgia [21,22].

Medical records were also reviewed to abstract age at study visit, sex, race, height and body weight, analgesic medication use (e.g., use of narcotic analgesics, non-steroidal anti-inflammatory drugs, acetaminophen, or gabapentin), medical comorbidities (i.e., diabetes mellitus, hypertension, congestive heart failure, coronary artery disease, hyperlipidemia, obstructive sleep apnea, chronic kidney disease, psoriasis, inflammatory bowel disease, lymphoma, thyroid disease, history of arthritis, anxiety disorder, depression), HIV-related data (CD4 cell count, HIV RNA level, use of antiretroviral therapy), HCV-related data (HCV genotype, HCV RNA level, current or prior interferon-based HCV therapy; hepatic decompensation diagnoses [ascites, spontaneous bacterial peritonitis, esophageal variceal hemorrhage, hepatic encephalopathy]; hepatocellular carcinoma), relevant social history (history of smoking, alcohol use, intravenous drug use, homelessness, incarceration, blood transfusion), and most recently recorded laboratory results of total bilirubin, asparate aminotransferase (AST), alanine aminotransferase (ALT), albumin, international normalized ratio (INR), white blood cell count, hemoglobin, and platelet count.

### Data analysis

The prevalence of joint pain was determined and expressed as a point estimate (percent) and 95% confidence interval (CI) within each group. Descriptive statistics were used to compare characteristics between groups (HIV/HCV-coinfected, HCV-monoinfected, and HIV-monoinfected). Differences between groups were determined using Chi-square or Fisher’s exact tests, when appropriate, for categorical data, and Wilcoxon rank-sum tests for continuous data. Logistic regression was used to determine adjusted odds ratios (aORs) with 95% CIs of joint pain associated with risk factors of interest among chronic HCV-infected (i.e., HIV/HCV-coinfected and HCV-monoinfected) and HIV-infected (i.e., HIV/HCV-coinfected and HIV-monoinfected) patients. Variables evaluated as risk factors for joint pain included age, sex, race, history of arthritis, history of anxiety disorder or depression, obesity (body mass index [BMI] > 30 kg/m^2^), AST, ALT, current HCV therapy, use of analgesic medications, HCV genotype (in chronic HCV-infected patients only), and CD4 cell count (in HIV-infected patients only). Since HCV treatment has been associated with arthralgias, we performed a sensitivity analysis in which we excluded patients currently utilizing this therapy and repeated the analyses above.

### Ethical approval

This study was approved by the Institutional Review Boards of the University of Pennsylvania and PVAMC. Written informed consent was obtained from all patients participating in the study.

## Results

A total of 202 participants were enrolled (79 HIV/HCV-coinfected; 93 HCV-monoinfected; 30 HIV-monoinfected). The characteristics of each group are presented in Table 1. Among the three groups, age and sex distributions were similar. Over half of participants were black (African-American or Caribbean-American decent). HCV genotype 1 was the most common genotype among chronic HCV patients. Median HCV RNA levels were higher in coinfected than HCV-monoinfected patients. Few chronic HCV-infected patients were currently receiving HCV therapy but approximately half previously received HCV treatment. Three patients reported a prior diagnosis of rheumatoid arthritis (2 with HCV monoinfection, 1 with HCV/HIV coinfection), one with systemic lupus erythematosus (HCV-monoinfected), one with ankylosing spondylitis (HIV-monoinfected), and one with inflammatory arthritis not otherwise specified (HCV-monoinfected). Use of analgesic medications was similar among the groups. The mean BMI was significantly lower in coinfected and HIV-monoinfected patients. Liver aminotransferase levels were higher and platelet counts were lower in coinfected and HCV-monoinfected patients. Other laboratory values did not significantly differ between the groups (Additional file 1: Table S1).

**Table 1:** **Baseline characteristics of human immunodeficiency virus (HIV)/chronic hepatitis C virus (HCV)-coinfected, chronic HCV-monoinfected, and HIV-monoinfected participants**

	HCV/HIV-	HCV-	HIV-	P-value*	
	Coinfected	Monoinfected	Monoinfected	HCV/HIV	HCV/HI
	N = 79	N = 93	N = 30	vs HCV	V vs HIV
**Site** (n, %)				<0.001	<0.001
HUP	2 (3%)	35 (38%)	0 (0%)		
PPMC	35 (44%)	20 (22%)	1 (3%)		
PVAMC	42 (53%)	38 (41%)	29 (97%)		
**Age** (Median, IQR)	55 (43–64)	57.5 (53.5-61)	54 (51–60)	0.04^†^	NS^†^
**Sex** (n, %)				NS	NS
Male	68 (86%)	77 (83%)	28 (93%)		
Female	11 (14%)	16 (17%)	2 (7%)		
**Race** (n, %)				0.001	NS
Caucasian	9 (11%)	35 (38%)	5 (17%)		
Black	64 (81%)	50 (54%)	24 (80%)		
Hispanic	4 (5%)	3 (3%)	0 (0%)		
Other	2 (3%)	2 (2%)	1 (3%)		
**BMI** (kg/m^2^)	25.0	29.2	27.2	<0.001^†^	0.05^†^
Median (IQR)	(22.5-29.0)	(25.5-33.2)	(25.0-33.2)		
**Current smoker** (n, %)	29 (37%)	37 (40%)	9 (30%)	NS	NS
**HCV Treatment** (n, %)				0.01	N/A
Current	4 (5%)	17 (18%)	0 (0%)		
Past	22 (28%)	31 (33%)	0 (0%)		
Never	53 (67%)	45 (48%)	30 (100%)		
**HCV Genotype** (n, %)				NS	N/A
1 (A or B)	70 (89%)	60 (65%)	0 (0%)		
2	1 (1%)	5 (5%)	0 (0%)		
3	1 (1%)	4 (4%)	0 (0%)		
**HCV RNA** (IU/mL)	1.37	0.62	N/A	0.02^†^	N/A
Median in million (IQR)	(0.44-4.63)	(0.03-2.70)			
**CD4 count** (cells/mm^3^)	474	N/A	480	N/A	NS^†^
Median (IQR)	(358–666)		(295–673)		
**Antiretroviral**	67 (85%)	0 (%)	26 (87%)	N/A	NS
**therapy** (n, %)					
**Previous arthritis diagnosis**^**‡**^ (n, %)	37 (40%)	28 (35%)	8 (27%)	NS	NS
**Analgesic medication use** (n, %)				NS	NS
Any	41 (52%)	49 (53%)	20 (67%)		
Opiate	15 (19%)	15 (16%)	4 (13%)		
Gabapentin	17 (22%)	11 (12%)	8 (27%)		
NSAIDs	21 (27%)	23 (25%)	10 (33%)		
Tylenol	10 (13%)	18 (19%)	9 (30%)		

*Abbreviations*: HUP = Hospital of University of Pennsylvania, PPMC = Penn Presbyterian Medical Center, PVAMC = Philadelphia Veterans Affairs Medical Center, BMI = body mass index, NS = Not significant (p > 0.05), N/A = Not Applicable.

^**‡**^Previous arthritis diagnosis included rheumatoid arthritis (N = 4), osteoarthritis (N = 46), ankylosing spondylitis (N = 1), systemic lupus erythematosus (N = 1), and patient reported “arthritis” but unknown type (N = 22). One patient carried more than one diagnosis.

^†^Wilcoxan-rank sum test, remainder of the p-values were calculated using the chi2 test.

Over half of the patients reporting joint pain also self-reported depression and anxiety on the MD-HAQ. Depression was reported by 50% of HCV-monoinfected, 80% of HIV-monoinfected, and 64% HIV/HCV-coinfected patients. Similarly, anxiety was reported by 51% of HCV-monoinfected, 67% of HIV-monoinfected, and 77% of coinfected patients.

Joint pain was more commonly reported among HCV-monoinfected than coinfected (71% versus 56%; p = 0.038) or HIV-monoinfected patients (71% versus 50%; p = 0.035). After adjustment for age and sex, HCV-monoinfected patients were 1.9-fold more likely to report joint pain compared to coinfected patients (aOR, 1.94; 95% CI, 1.02-3.67) and 2.4-fold more likely to report arthralgias compared to HIV-monoinfected patients (aOR, 2.43; 95% CI, 1.03-5.72). No differences in the prevalence of joint pain were observed between coinfected and HIV-monoinfected patients (56% versus 50%; p = 0.59). HIV/HCV coinfection remained unassociated with arthralgias compared to HIV monoinfection after adjustment for age and sex (aOR, 1.25; 95% CI, 0.54-2.92). A sensitivity analysis excluding chronic HCV-infected patients currently on HCV therapy (n = 22) revealed similar proportions of chronic HCV-infected persons reporting joint pain (data not shown).

**Figure 1:**
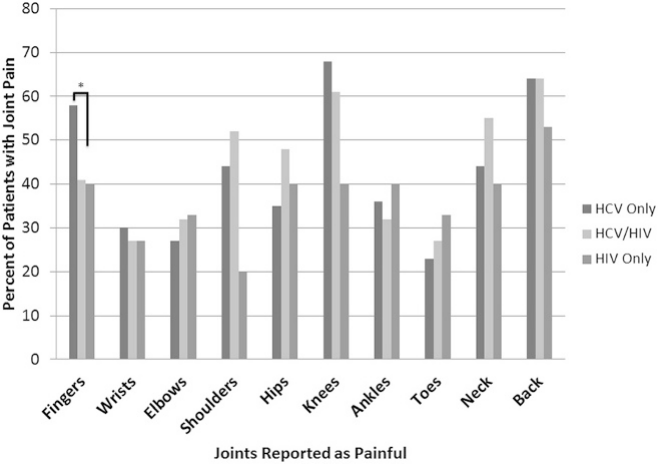
Sites of joint pain, by human immunodeficiency virus (HIV) and chronic hepatitis C virus (HCV) Status (%).The distribution of painful joints was similar among the groups, although HCV-monoinfected patients more commonly reported finger pain compared to coinfected (41% versus 27%; p = 0.035) and HIV-monoinfected (41% versus 23%; p = 0.067) patients. The p-values for the remainder of the comparisons were >0.05.

Among all three groups, fingers, knees, and back were the most frequently cited areas of joint pain (Figure 1). The distribution of painful joints was similar among the groups, although HCV-monoinfected patients more commonly reported finger pain compared to coinfected (41% versus 27%; p = 0.035) and HIV-monoinfected (41% versus 23%; p = 0.067) patients.

MD-HAQ results among participants reporting joint pain are reported in Table 2. The mean (SD) MD-HAQ among participants reporting joint pain was 6.0 (2.77), and the mean (SD) RAPID3 was 12.9 (5.46). The RAPID3 components did not significantly differ by HIV/HCV status. The mean (SD) duration of morning stiffness among participants reporting joint pain was 57.3 (158.1) minutes. Symptoms of dry mouth, ascertained by the MD-HAQ, were less commonly reported among HIV-monoinfected than coinfected (20% versus 45%; p = 0.08) or HCV-monoinfected (20% versus 48%; p = 0.04) patients. Coinfected participants were more likely to report myalgias than HCV-monoinfected (43% versus 27%; p = 0.06) or HIV-monoinfected (43% versus 7%; p = 0.01) persons. Additionally, patients with HIV/HCV coinfection were more likely to report numbness or tingling of the extremities compared to patients with HCV monoinfection (41% versus 27%; p = 0.14) or HIV monoinfection (41% versus 7%; p = 0.01).

**Table 2:** **Multidimensional Health Assessment Questionnaire results among participants reporting joint pain**

	HCV/HIV-	HCV-	HIV-
	Coinfected	Monoinfected	Monoinfected
Physical Function Score (Mean(SD))	2.4 (2.0)	2.6 (1.5)	3.3 (2.1)
Pain (Mean(SD))	6.5 (2.7)	5.6 (2.7)	6.2 (3.2)
Patient Global (Mean(SD))	3.9 (3.3)	4.4 (2.4)	4.9 (1.7)
RAPID3 (Mean(SD))	12.8 (6.3)	12.6 (4.9)	14.3 (5.6)
Emotional Score*(Mean(SD))	4.2 (2.7)	3.7 (2.4)	3.7 (2.3)
Total Symptoms (Mean(SD))**	18.6 (10.4)	17.6 (9.8)	14.2 (10.8)
Depression, n (%)	28 (64%)	33 (50%)	12 (80%)
Anxiety, n (%)	34 (77%)	34 (51%)	10 (67%)
Sleep Disturbance, n (%)	32 (73%)	41 (62%)	10 (67%)
Dry Eyes, n (%)	6 (14%)	19 (29%)	0 (0%)
Dry Mouth n (%)	20 (45%)	32 (48%)	3 (20%)
Numbness/tingling n (%)	18 (41%)	18 (27%)	1 (7%)
Myalgias, n (%)	19 (43%)	17 (26%)	1 (7%)
Morning Stiffness, n (%)	29 (66%)	44 (67%)	8 (53%)
Fatigue Scale (Mean(SD))	4.53 (3.68)	4.73 (3.44)	3.97 (3.82)
≥20 Symptoms Reported	42 (45%)	43 (54%)	7 (23%)

Only patients reporting joint pain completed the MD-HAQ. *The MD-HAQ includes an emotional function assessment in which depression, anxiety and sleep disturbance each ranked as none (0), mild (1.1), moderate (2.2) or severe (3.3). The total “emotional score” is the combined sum of these three components (range 0–9.9).

**The MD-HAQ contains a 60-item review of systems (including depression, anxiety, sleep disturbance, dry eyes, dry mouth, numbness/tingling, myalgias, and morning stiffness. There is additionally a visual analog scale for fatigue.

A previous diagnosis of arthritis (aOR, 4.25; 95% CI, 1.84-9.81) and current smoking (aOR, 5.02; 95% CI, 2.15-11.74) were risk factors for self-reported joint pain among chronic HCV-infected patients (Table 3). These associations remained when persons currently receiving HCV therapy were excluded (data not shown). Among HIV-infected patients, current smoking (aOR, 6.07; 95% CI, 2.30-16.00) and a previous diagnosis of arthritis (aOR, 5.36; 95% CI, 2.01-14.25) were also associated with arthralgias.

**Table 3:** **Factors associated with joint pain in chronic hepatitis C virus-infected and human immunodeficiency virus-infected participants**

	HCV		HIV	
	Univariable models (Unadjusted)	Multivariable model	Univariable models (Unadjusted)	Final model
	OR (95% CI)	OR (95% CI)	OR (95% CI)	OR (95% CI)
Co-infection	0.51 (0.27-0.97)	0.49 (0.23-1.05)	1.26 (0.54-2.92)	
Age (cont)	1.01 (0.96-1.06)		0.97 (0.91-1.03)	
Female Sex*	0.95 (0.41-2.23)		1.41 (0.43-4.63)	
Current Smoker	3.50 (1.71-7.18)	5.02 (2.15-11.74)	5.43 (2.18-13.52)	6.07 (2.30-16.00)
Obesity (BMI > 30)	1.85 (0.90-3.79)	1.29 (0.57-2.92)	1.70 (0.68-4.29)	
Previous Diagnosis of Arthritis†	4.47 (2.11-9.48)	4.25 (1.84-9.81)	4,74 (1.90-11.81)	5.36 (2.01-14.25)

*Males are the referent group. †Previous arthritis diagnosis included rheumatoid arthritis (N = 4), ankylosing spondylitis (N = 1), systemic lupus erythematosus (N = 1), osteoarthritis (N = 46), and patient reported “arthritis” but unknown type (N = 22). Abbreviations: BMI = body mass index.

## Discussion

This cross-sectional study demonstrated that joint pain was commonly reported and associated with diminished functional status and emotional well-being among patients with chronic HCV and/or HIV infection. The prevalence of joint pain was significantly higher among HCV-monoinfected than HIV/HCV-coinfected and HIV-monoinfected patients. Over two-thirds of patients with chronic HCV monoinfection reported joint pain, while approximately half of coinfected and HIV-monoinfected patients reported arthralgias.

Our study extends results of prior cross-sectional analyses, which have reported that 67-81% of chronic HCV-infected patients complain of musculoskeletal pain [23,24]. Tsui et al. [25] found that HIV/HCV coinfection was more commonly associated with musculoskeletal pain compared to HIV-monoinfection (aOR, 1.45; 95% CI, 1.06-1.97), and adjusting for inflammatory cytokine levels and depression did not change these results. Consistent with our results, Cacoub et al. [26] found that HIV/HCV-coinfected patients less commonly had arthralgias compared with HCV-monoinfected patients (5% versus 29%). However, none of these studies directly compared joint complaints among HIV/HCV-coinfected, HCV-monoinfected, and HIV-monoinfected patients, as was performed in this study.

The etiology of joint pain in chronic HCV infection remains unclear. Immune activation, direct viral particle deposition in the synovium, and the high prevalence of concomitant mood disorders might be important contributors to the arthralgias commonly reported by chronic HCV-infected patients [10,27,28].

The differences in the prevalence of joint pain among the three study groups raise important questions for further study. Hypothesized explanations for these findings include differences in unmeasured environmental exposures among the groups or potentially a decrease in local inflammation related to immune dysfunction in HIV infection, resulting in a decreased effect of chronic HCV in the HIV/HCV-coinfected patients [29,30] Additionally, HIV-infected patients may be more tolerant of joint discomfort due to higher priority placed on more serious complications of their underlying disease. In general, HCV-infected patients with joint pain had a high prevalence of self-reported depression, anxiety, and sleep disturbance. Depression and anxiety could have contributed to a higher prevalence of joint pain reported by this group since mood and joint pain are strongly linked.

We found that current smoking was strongly associated with joint pain. Smoking has been associated with musculoskeletal pain and the development of rheumatoid arthritis [31] In addition, smoking is associated with worse disease activity in patients with rheumatoid arthritis [32]. However, smoking and its link to joint pain in patients with chronic HCV has not previously been reported. Importantly, smoking is a modifiable risk factor. Further research is needed to determine whether joint pain in patients with chronic HCV improves with smoking cessation.

7We found that nearly half of patients with chronic HCV infection (both HCV-monoinfected and HIV/HCV-coinfected) and joint pain self-reported 20 or more symptoms which has previously been reported to indicate a fibromyalgia diagnosis [33]. Fibromyalgia has been reported to be common among patients with chronic HCV although the reason for this association is unclear [27,28].

Our study was limited by its cross-sectional design, use of convenience sampling, our inability to perform physical examinations and obtain imaging studies to rule out osteoarthritis, and the relatively small sample. In particular, there is the possibility that patients with joint pain may have been more likely to complete the survey. Additionally, we were unable to determine the specific etiologies for the joint pain. While many patients reported a diagnosis of arthritis, we did not confirm these diagnoses on physical examination or further testing. Furthermore, some patients were prescribed analgesic medications, and these patients might have less commonly self-reported joint pain. Finally, our study evaluated patient-reported joint complaints present within the one week prior to the survey date. Thus, there may have been patients with intermittent joint pain who were not identified in this study. However, use of the one week time frame limits the risk of recall bias and is the time period specified on the MD-HAQ for assessment of disease activity in rheumatoid arthritis.

## Conclusions

Among patients with HIV and/or chronic HCV infections, joint pain was prevalent. HCV-monoinfected patients more frequently reported arthralgias compared to HIV/HCV-coinfected or HIV-monoinfected persons. These results suggest that joint pain remains a major health concern and a determinant of health-related quality of life among these patients. Providers should seek to address modifiable risk factors for joint pain (e.g., smoking cessation) in such persons. Future studies should determine the etiologies and strategies for management of joint pain, including fibromyalgia, in patients with chronic HCV and HIV infection, as well as the mechanisms for modulation of joint symptoms in HIV/HCV-coinfected patients.

## Additional file

Additional file 1: Table S1. Laboratory results of human immunodeficiency virus (HIV)/chronic hepatitis C virus (HCV)-coinfected, chronic HCV-monoinfected, and HIV-monoinfected participants.

## Abbreviations

ALT: Alanine aminotransferase
AST: Asparate aminotransferase
BMI: Body mass index
HCV: Hepatitis C Virus
HIV: Human Immunodeficiency Virus
HUP: Hospital of the University of Pennsylvania
INR: International normalized ratio
IQR: Interquartile range
MD-HAQ: Multi-Dimensional Health Assessment Questionnaire
NSAID: Non-steroidal anti-inflammatory drug
PPMC: Penn Presbyterian Medical Center
PVAMC: Philadelphia Veterans Affairs Medical Center
RAPID3: Routine Assessment of Patient Index Data
RNA: Ribonucleic acid
