# Supplementary material for: Prevalence and risk factors for patient-reported joint pain among patients with HIV/Hepatitis C coinfection, Hepatitis C monoinfection, and HIV monoinfection
Source: BMC Musculoskelet Disord. 2015 Apr 19;16:93. doi: 10.1186/s12891-015-0552-z (PMC4404567; doi:10.1186/s12891-015-0552-z)
Supplement: Supplementary file 1 — Laboratory results of human immunodeficiency virus (HIV)/chronic hepatitis C virus (HCV)-coinfected, chronic HCV-monoinfected, and HIV-monoinfected participants. [file 12891_2015_552_MOESM1_ESM.docx]

**Supplemental Table 1: Laboratory results of human immunodeficiency virus (HIV)/chronic hepatitis C virus (HCV)-coinfected, chronic HCV-monoinfected, and HIV-monoinfected participants.**

|  | **HCV/HIV-Coinfected**  **(n=79)**  Median (IQR) | **HCV-Monoinfected**  **(n=93)**  Median (IQR) | **HIV-**  **Monoinfected**  **(n=30)**  Median (IQR) | **P-value*** | |
| --- | --- | --- | --- | --- | --- |
|  |  |  |  | **HCV/HIV vs. HCV** | **HCV/HIV vs. HIV** |
| AST (U/L) | 44 (31-67) | 54.5 (34-85) | 25 (22-32) | NS | <0.001 |
| *Missing (n [%])* | *1 (1%)* | *9 (10%)* | *0 (0%)* |  |  |
| ALT (U/L) | 38.5 (25-59) | 49.5 (31.5-75.5) | 22.5 (18-31) | 0.05 | <0.001 |
| *Missing (n [%])* | *1 (1%)* | *9 (10%)* | *0 (0%)* |  |  |
| Alkaline Phosphatase (U/L) | 84 (65-121) | 83.5 (63-113) | 84 (71-113) | NS | NS |
| *Missing (n [%])* | *1 (1%)* | *11 (12%)* | *0 (0%)* |  |  |
| Albumin (g/dl) | 3.9 (3.55-4.25) | 3.9 (3.3-4.3) | 4.1 (3.7-4.4) | NS | NS |
| *Missing (n [%])* | *3 (3%)* | *14 (15%)* | *0 (0%)* |  |  |
| Creatinine (mg/dl) | 1.05 (0.89-1.3) | 0.96 (0.84-1.14) | 1.05 (0.96-1.3) | 0.02 | NS |
| *Missing (n [%])* | *1 (1%)* | *10 (11%)* | *0 (0%)* |  |  |
| Sodium (mmol/dl) | 138 (137-140) | 139 (137-140) | 139 (136-140) | NS | NS |
| *Missing (n [%])* | *12 (15%)* | *20 (22%)* | *0 (0%)* |  |  |
| Platelets (thou/µl) | 177 (131-242) | 153 (95-209) | 207 (174-261) | 0.05 | 0.03 |
| *Missing (n [%])* | *0 (0%)* | *14 (15%)* | *0 (0%)* |  |  |
| Hemoglobin (g/dl) | 13.6 (12.1-14.8) | 13.6 (12.6-14.8) | 13.75 (12.6-14.6) | NS | NS |
| *Missing (n [%])* | *0 (0%)* | *12 (13%)* | *0 (0%)* |  |  |
| White Blood Cells (thou/µl) | 5.1 (4-6.8) | 5.75 (3.95-7.3) | 5.85 (4.4-6.8) | NS | NS |
| *Missing (n [%])* | *0 (0%)* | *13 (14%)* | *0 (0%)* |  |  |
| INR | 1.06 (1-1.12) | 1.09 (1-1.2) | 1.04 (0.99-1.1) | NS | NS |
| *Missing (n [%])* | *19 (24%)* | *27 (29%)* | *2 (7%)* |  |  |
| Abbreviations: AST=aspartate aminotransferase, ALT=alanine aminotransferase, INR=International Normalized Ratio, NS= not significant (p>0.05).  P-values for all comparisons were calculated using Wilcoxan rank-sum tests. | | | | | |
